# Efficacy and Safety of Ganduqing Granules in Treating the Common Cold: A Multicenter, Randomized, Double-Blind, Placebo-Controlled Trial

**DOI:** 10.1155/2022/5105503

**Published:** 2022-06-09

**Authors:** Yilan Wang, Piao Zhou, Yuxiao Wu, Huaqin Cao, Wenfeng Hao, Fei Wang, Jing Guo

**Affiliations:** ^1^Hospital of Chengdu University of Traditional Chinese Medicine, Chengdu 610072, Sichuan, China; ^2^Community Health Service Center of Simaqiao, Chengdu 610084, Sichuan, China

## Abstract

**Background:**

There is no clear evidence-based medicine that points to the most effective drug treatments for the common cold. In view of its ability to relieve symptoms and shorten the disease course, as well as its minimal side effects, traditional Chinese medicine (TCM) has been widely used to treat the common cold. However, there is a lack of strong evidence to support the clinical efficacy of TCM. This study aimed to evaluate the efficacy and safety of Ganduqing granules in the treatment of the common cold based on the network pharmacology analysis.

**Methods:**

In this multicenter, randomized, double-blind, placebo-controlled trial, 60 eligible subjects will be randomly assigned to either the intervention group or the placebo group. The intervention group will be treated with Ganduqing granules, while the placebo group will be treated with placebo. After 5 days of intervention, the efficacy and safety of Ganduqing granules in the treatment of the common cold will be observed. The primary outcome is the time to clearance of all symptoms. The secondary outcomes included the levels of IL-6, TNF-*α*, SOD, and MDA in the peripheral blood, time to disappearance of primary symptoms and secondary symptoms, clinical symptom remission rate, and change in TCM syndrome score.

**Results:**

Sixty participants completed the study. Ganduqing granules showed a greater effect on the time to clearance of all symptoms (*P* < 0.0001), nasal discharge (*P*=0.0124), fatigue and lack of strength (*P*=0.0138), dryness of the pharynx (*P* < 0.0001), pharyngalgia or dysphagia (*P* < 0.0001), and expectoration (*P* < 0.0431) compared with the placebo group. Participants in the intervention group had a greater decrease of IL-6 levels compared with the placebo group (*P* < 0.007); similar results were observed for the SOD (*P* < 0.033). However, the change in TNF-*α* and MDA levels in the intervention group was not significantly different from the placebo group. In addition, participants in the intervention group had a greater decrease of TCM syndrome score compared with the placebo group (*P* < 0.040).

**Conclusion:**

Ganduqing granules could improve common cold symptoms, shorten the disease course, attenuated inflammation and oxidative stress, and provided objective evidence for the efficacy and safety of a Chinese herbal medicine in treating the common cold.

## 1. Introduction

The common cold is a frequently occurring disease principally caused by viruses that belong to the category of mild acute upper respiratory tract infection [1]. It is a highly prevalent condition experienced by humans, and it has a significant impact on society and health care [2]. More than 200 viruses are associated with the common cold, with rhinovirus being the most common [3]. According to statistics from the United States, approximately 25 million people suffer from the common cold each year, with adults experiencing the common cold on average 4–6 times per year and children 6–8 times per year [4]. According to a study in the United States, 30% of cases of absenteeism from work and 40% of cases of absenteeism from education are caused by the common cold [5, 6]. The treatment of complications and the deterioration of primary disease caused by the common cold are responsible for a significant increase in medical costs and a marked overall disease burden [7]. Despite the high incidence and economic impact of the common cold, no effective treatment has been found [8]. Treatments to date have focused on symptom relief, and although the effectiveness of Western medicine in the treatment of the common cold has improved, there is still no established medication that targets pathogens to cure the condition. At present, the drugs commonly used to treat the common cold in Western medicine mainly include antipyretic and analgesic drugs, antihistamines, decongestants, cough medicines, expectorants, vitamins, and probiotics, among others (Table 1). However, the effectiveness of commonly used drugs is unclear, and they are associated with many side effects. Moreover, to date, no evidence-based guidelines for the common cold have been published in clinical practice. Furthermore, patients often buy antibiotics themselves when suffering from the common cold, which leads to antibiotic abuse and other adverse consequences [35].

Traditional Chinese medicine (TCM) has a long history as a holistic system of medicine, integrating prevention, treatment, and rehabilitation. Because of the simplicity and effectiveness of TCM in treating the common cold, many TCM doctors, as well as the public in China, seem to agree that TCM is effective in reducing symptoms and shortening the duration of the common cold. Ganduqing granules, a Chinese herbal medicine composed of Radix Astragali (Huangqi) and Rhizoma Belamcandae (Shegan) for the treatment of the common cold, were approved for clinical trials by the China Food and Drug Administration. In preliminary clinical trials, Ganduqing granules were effective in relieving the symptoms of the common cold, especially throat symptoms, [36–38] with few adverse reactions and good safety.  Table 2 summarizes the main components in recent studies on the pharmacological effects of Ganduqing granules.

**Figure 1:**
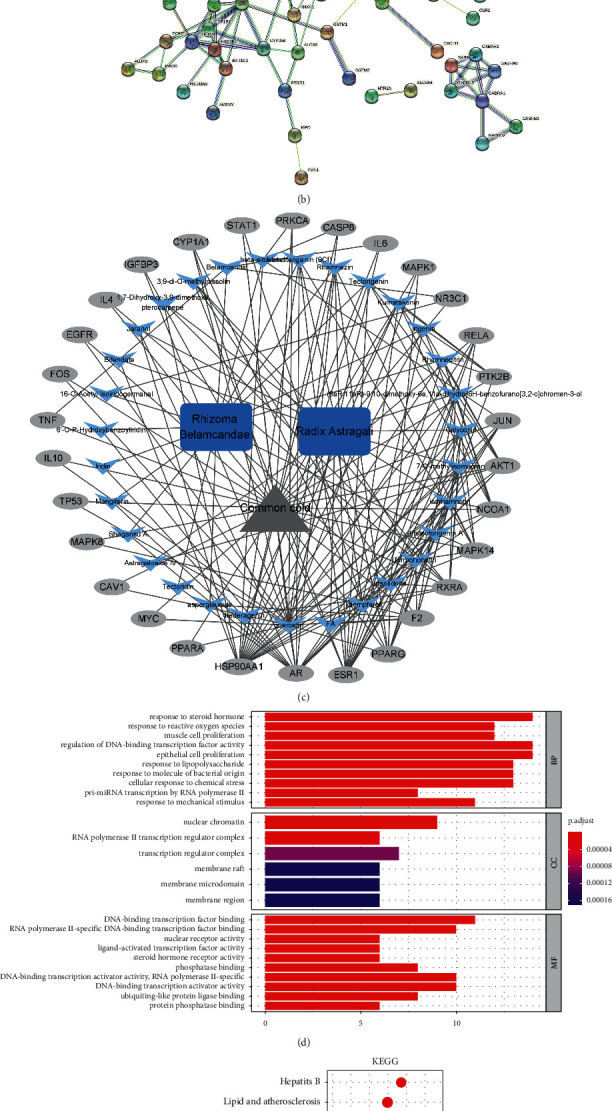
Network pharmacology of Ganduqing for the treatment of common cold. (a) Venn diagram shows intersection of gene sets. (b) The protein-protein interaction (PPI) network. (c) The drug-target-disease network. (d) The Gene Ontology (GO) analysis results. (e) The KEGG pathway analysis results.

Network pharmacology is a new method of drug research that shows the relationship between drug and diseases [47]. The data collected by network pharmacology indicated that 246 target genes of Ganduqing for the treatment of common cold were found (Figure 1(a)). In addition, the protein-protein interaction network among the target genes is shown in Figure 1(b). At the same time, a network of drugs, drug active ingredients, target genes corresponding to the active ingredients, and disease targets was constructed (Figure 1(c)). The GO enrichment analysis results of Ganduqing granules for the treatment of common cold are shown in Figure 1(d). The biological process mainly involved the response to steroid hormones, response to reactive oxygen species, and muscle cell proliferation; cellular components primarily involved nuclear chromatin, transcription regular complex, and membrane raft; molecular function mainly involved DNA-binding transcription factor binding, nuclear receptor activity, and ligand-activated transcription factor activity. The results of KEGG enrichment analysis indicated that the signaling pathways involved in the treatment of common cold by Ganduqing granules mainly included the IL-17 signaling pathway, Toll-like receptor signaling pathway, C-type lectin receptor signaling pathway, T cell receptor signaling pathway, and TNF signaling pathway (Figure 1(e)).

At present, there is insufficient evidence to support the efficacy of Ganduqing granules in patients with the common cold. Our aim is to conduct a multicenter, randomized, double-blind, placebo-controlled trial to evaluate the efficacy and safety of Ganduqing granules in patients with the common cold.

## 2. Methods

### 2.1. Study Design

The trial has been approved by the Medical Ethics Committee of the Hospital of Chengdu University of Traditional Chinese Medicine (approval No. 2019KL-044) and has been registered with Clinical trial registration in China: ChiCTR1900026714 (registration date: October 19, 2019. https://www.chictr.org.cn/showproj.aspx?proj=44531).

This study is a multicenter, randomized, double-blind, placebo-controlled trial. Sixty eligible subjects were randomly assigned to either the intervention group or the placebo group. After 5 days of intervention, the effectiveness and safety of Ganduqing granules were evaluated by comparing the primary and secondary outcomes between the two groups.

The participating units included the Hospital of Chengdu University of Traditional Chinese Medicine, West China Hospital of Sichuan University, Third People's Hospital of Chengdu, and Community Health Service Center of Simaqiao, Chengdu. The four centers are all located in Chengdu, Sichuan Province, China, and each center had a principal investigator responsible for conducting clinical trials and collecting relevant data. As the leading unit of the study, the Hospital of Chengdu University of Traditional Chinese Medicine was responsible for training the researchers on standard operating procedures and supervising the research progress of all clinical subcenters. Participants were recruited by competitive enrollment at all the centers. All participants were required to provide written informed consent before entering the trial. An independent data and safety monitoring board (DSMB) monitored the conduct and safety of the trial to ensure patient safety. Additionally, the study was implemented in accordance with the Declaration of Helsinki [48] (2013) and the Guidelines for Good Clinical Practice [49]. The trial was strictly designed in accordance with the CONSORT statement [50].

### 2.2. Participant Recruitment

We enrolled participants through advertising and recommendations. The trial began in October 2019 and was finished in March 2021. Patients with the common cold of Qi-deficiency syndrome were enrolled. Qi-deficiency is a typical syndrome observed in TCM. In the Guidelines for Diagnosis and Treatment of Common Cold in Traditional Chinese Medicine, [51] the common cold of Qi-deficiency syndrome is defined as follows—primary symptoms: aversion to wind and cold, fever, nasal congestion, nasal discharge, fatigue and lack of strength, dyspnea and reluctancy to speak, dryness of the pharynx, pharyngalgia, or dysphagia; secondary symptoms: headache, limb soreness, cough, expectoration; tongue appearance: pale tongue, yellow and thin fur, white and thin fur; and pulse condition: superficial or sunken pulse. Participants should have at least three primary symptoms, one secondary symptom, and any of the above tongue appearance and pulse condition to be diagnosed with the common cold of Qi-deficiency syndrome.

### 2.3. Inclusion Criteria

The study inclusion criteria were as follows: (1) fulfillment of the diagnostic criteria for the common cold in Western medicine [52, 53]; (2) compliance with the criteria for Qi-deficiency syndrome in TCM [51]; (3) acute onset of illness within 48 h (no antibiotics or other drugs for the treatment of common cold); (4) aged between 18 and 65 years; and (5) voluntary written informed consent.

### 2.4. Exclusion Criteria

The exclusion criteria were as follows: (1) influenza, herpetic pharyngitis, suppurative tonsillitis, or pulmonary infection; (2) severe primary diseases, such as those of the heart, brain, or hematopoietic system; (3) abnormal liver function (such as an alanine aminotransferase or aspartate aminotransferase concentration 1.5 times the normal upper limit), abnormal serum creatinine, or a positive qualitative urine protein test; (4) malignant tumors; (5) mental illness; (6) body temperature (underarm) of >39.0°C; (7) peripheral white blood count of >12.0 × 10^9^/L and a peripheral neutrophil percentage of >80%; (8) any medication to relieve common cold symptoms; (9) pregnancy/lactation or a birth plan; (10) a prior history of allergy to the drug components used in this clinical trial; (11) participation in other clinical trials in the past month; or (12) judged by the investigator as inappropriate for participation in the clinical trial.

### 2.5. Sample Size

Participants were randomly divided into the intervention group and the placebo group. The time to clearance of all symptoms—the length from study enrollment to the time when the symptoms completely disappear—was used to calculate the sample size of the trial. According to our previous work, the average duration of common cold symptoms is 78.62 ± 28.07 hours, and the mean duration of common cold symptoms decreased by 32.56 hours after Ganduqing granules intervention. The number of cases required in each group is estimated to be 25 based on 90% weight (1 − *β*) and *α* = 0.05 (bilateral) using PASS, version 11 (NCSS, LLC, Kaysville, UT, USA). To determine the required number of participants, based on a shedding rate of 20%, a total of 60 participants were included in the trial.

### 2.6. Randomization and Blinding

In this study, the double-blind randomized method was adopted. We used the block randomization method with the block length of 8. SAS 9.2 software (SAS Institute Inc., Cary, NC, USA) was used to generate a random sequence of 60 participants, with the serial number 01–60. A random number card was filled in by a professional statistician according to the assignment plan and sealed in an opaque envelope. Participants were recruited by clinical investigators, and when participants were admitted into the group, third-party staff opened the envelopes according to their entry order. Participants were grouped and distributed drugs according to the random card number in the envelope. Blinding was set through SAS 9.2 software by a professional statistician. The first level of blinding represented the corresponding serial number for the two groups, and the second level of blinding represented the corresponding intervention measures for the intervention group and placebo group. After the end of the trial, the data analysis staff performed the first level of unblinding to reveal the groups corresponding to each serial number for data statistics. When statistics were complete, the researchers performed the second level of unblinding to reveal the intervention used in each group. Patients, clinical investigators, and data analysis staff were blinded during trial implementation, data collection, and data analysis. The envelope was properly kept until the end of the study. Each participant was equipped with an opaque emergency envelope containing the information on the participant's random number and the matching treatment group. Once the envelope is opened, the corresponding subject will be deemed to have been unblinded.

### 2.7. Interventions

After participants enrolled in the trial have provided written informed consent, baseline measurements were taken by clinical researchers. Third-party staff administered drugs according to the random card number. The intervention group was given Ganduqing granules (Ganduqing granules have been condensed into 6 g/bag; batch number: 1902072), and the placebo group was administered placebo. Placebo and Ganduqing granules have been prepared by Sichuan Neo-Green Pharmaceutical Technology Development Co. Ltd., Sichuan, China, which has a China Pharmaceutical Production License. The drugs were prepared in accordance with the standards of good manufacturing practice. The placebo compound was made of starch and maltodextrin and did not contain any active ingredient. It was identical to Ganduqing granules in size, color, shape, and packaging. The number, usage, dosage, function, indications, manufacturer, storage conditions, and expiry date were clearly marked on the outer packaging, and a “trial use” label was attached.

Participants were instructed to dissolve the granules in 100 ml of boiled water and to take the solution orally at a temperature of between 30°C and 37°C, three times daily for 5 days (each package contains a 6-day supply). During treatment, participants should avoid smoking, drinking, and overworking. Participants were allowed to continue taking medication for chronic diseases, such as high blood pressure and diabetes mellitus. The dose, duration, and name of any concomitant medication or treatment were carefully recorded in the case report form (CRF). Any other therapies or drugs that may influence the results of the study were prohibited during the trial period. After 5 days, participants returned the remaining medication and its outer packaging. If the illness worsens or serious adverse events occur, the clinical trial will be terminated immediately, and the cause and associated outcome measures will be recorded in the CRF. The study program details are shown in Supplementary File 1.

### 2.8. Primary Outcome

The primary outcome included the time to clearance of all symptoms, which is defined as the time from study enrollment to complete disappearance of symptoms. Participants were instructed to record any change in symptoms in their participant diary. Investigators used the data to assess the duration of illness. The time to clearance of all symptoms was used as a measure of disease severity, with a longer time to clearance indicating more severe illness.

### 2.9. Secondary Outcomes

Secondary outcomes included the levels of IL-6, TNF-*α*, SOD, and MDA in the peripheral blood, time to disappearance of primary symptoms and secondary symptoms, clinical symptom remission rate, and change in TCM syndrome score [51].

The remission rate of all clinical symptoms was equal to the number of all symptom remission cases ÷ total number of cases × 100%. The sum of all symptom scores was the cumulative TCM symptom score. The TCM syndrome score was formulated in accordance with the guiding principle of clinical research on new drugs of traditional Chinese medicine (2002) [51, 54]. All symptoms were given graded scores according to whether they are mild, moderate, or severe. Tongue appearance and pulse condition were recorded separately without scoring. The specific scoring rules are shown in Supplementary File 2.

A change in the cumulative TCM symptom score was evaluated by the percentage symptom reduction, which was calculated according to the following formula: (scores before treatment − scores after treatment) ÷ scores before treatment × 100%.

“Clinical recovery” was indicated by disappearance of TCM clinical symptoms and signs (or approximate disappearance) and a decrease in TCM score of ≥90%. “Markedly effective” was indicated by a significant improvement in TCM clinical symptoms and signs and 70% ≤ syndrome score reduction rate < 90%. “Effective” was indicated by an improvement in TCM clinical symptoms and signs and 30% ≤ syndrome score reduction rate < 70%. Finally, “invalid” was indicated by no significant improvement in (or exaggeration of) TCM clinical symptoms or signs and a reduction in TCM score of <30%.

### 2.10. Safety Assessment

The safety outcomes included records of adverse events, physical examinations, and laboratory test results. Physical examinations included measurements of body temperature, respiration rate, blood pressure, and heart rate. Laboratory indicators included routine blood tests, routine urine tests, routine stool tests, liver function tests, renal function tests, chest X-rays, and electrocardiography examinations. These indicators were monitored at baseline and on the sixth day after treatment beginning. If the results of chest X-ray are normal before treatment, chest X-ray will not be performed during follow-up. Any adverse events that occur during the study period were carefully observed and recorded.

### 2.11. Adverse Events and Harm Reporting

For adverse events that occurred during the test, the symptoms, signs or laboratory examination results, occurrence time, duration, degree, treatment measures, and process were recorded in the CRF to evaluate their correlation with the study drugs, and the researchers signed and dated them.

When adverse reactions are detected, the researchers will propose treatment measures according to the illness and decide whether to suspend the study. In case of serious adverse events, necessary measures will be taken immediately to protect the safety of the participants.

### 2.12. Statistical Analysis

In this study, data analysis was conducted by dedicated data analysis staff who were blinded to the whole trial. They used SAS, version 9.2, and GraphPad Prism 8.12 software.

The intention-to-treat (ITT) analysis includes all participants after randomization, but complete follow-up of all participants may be difficult. The full analysis set (FAS) is a data set derived from all randomized participants who received at least 1 dose of the study drug. In accordance with the ITT principle in the statistical analysis of clinical trials, the FAS was used for statistical analysis. When case shedding results in missing data, the value of the last observation will be carried forward. Measurement data were expressed as mean ± standard deviation. The normality test was performed using the Kolmogorov–Smirnov test. Chi-squared tests were used to compare clinical symptom remission rates between groups. Time to clearance of all symptoms and time to disappearance of primary symptoms and secondary symptoms were compared by using *t*-tests. Given that the measurements of levels of IL-6, TNF-*α*, SOD, and MDA in the peripheral blood and change in TCM syndrome score were done before and after treatment, analysis of covariance methodology was used to perform statistical analysis. Prior to statistical analysis, the difference between the values measured before and after treatment was calculated; and the difference served as a dependent variable. The baseline measurement (IL-6, TNF-*α*, SOD, MDA, and TCM syndrome score), age, body weight, body temperature, respiratory rate, heart rate, time from onset to enrollment, and participating centers were taken as covariates for analyses. A *p* < 0.05 was considered statistically significant. All items in the CRF were accurately recorded and sent to the designated statistical center. All data were checked and entered by two data managers to ensure data accuracy.

### 2.13. Data Management

Case reports were written by clinical investigators at the centers involved in the trial. There was a complete case report for each participant and were checked by a supervisor. The paper version of the CRF was digitized for data entry and management by a dedicated data manager. All data were stored on a secure server with two backup copies on an external hard drive. Password-protected spreadsheets stored on secure servers linked the names and identification codes of all participants for re-identification if needed. All participants' personal information was kept confidential and was used for this study only. An independent DSMB, consisting of two clinical experts and one statistician, conducted a blind check on the study data every 6 months during study implementation to ensure safety and effectiveness are observed in participants' trial data.

### 2.14. Quality Control

The clinical study was discussed and revised many times by clinicians, statisticians, and methodologists before the study begins. All investigators involved in the trial were required to participate in study training to ensure that the study protocol and standard operating procedures are fully understood, such that the clinical study is conducted rigorously and properly and that the clinical data are accurate and complete. Each trial center had a principal investigator who was responsible for the quality of the research. This principal investigator was appropriately qualified and trained prior to the start of the trial. The pharmaceutical ingredients of Ganduqing granules were compliant with the standards of good manufacturing practice. Ganduqing granules were prepared by Sichuan Neo-Green Pharmaceutical Technology Development Co. Ltd., Sichuan, China. These drugs were under the control of the Hospital of Chengdu University of Traditional Chinese Medicine.

## 3. Results

### 3.1. Baseline Characteristics

**Figure 2:**
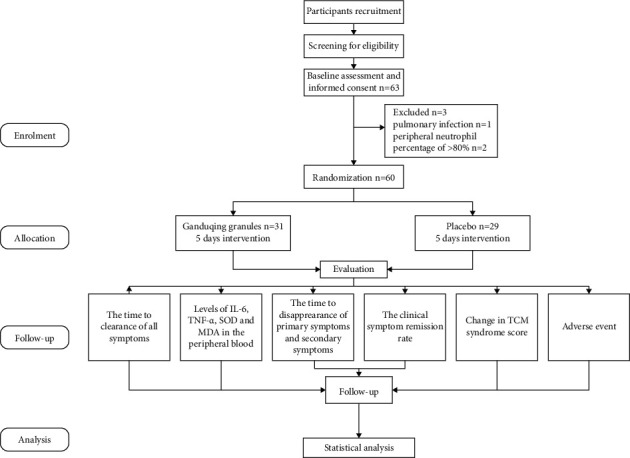
Flowchart of the study.

Between October 2020 and March 2021, 63 participants were screened and 3 participants were excluded for the laboratory test results did not met the inclusion criteria. The flowchart of participants through the trial is shown in Figure 2. The remaining 60 participants were randomly assigned to an intervention group or a placebo group, and the baseline characteristics of participants are shown in Table 3. No significant differences between the groups were observed in baseline characteristics including age, sex, body mass index, height, weight, body temperature, breath, heart rate, diastolic pressure, systolic pressure, time from onset to enrollment, levels of IL-6, TNF-*α*, SOD, and MDA in the peripheral blood, and TCM syndrome score.

#### 3.1.1. Patient Withdrawal

No participants dropped out during the study. Thus, a total of 60 participants completed the study without major protocol violations and had a compliance rate of 100%.

#### 3.1.2. The Time to Clearance of All Symptoms

Ganduqing granules showed a greater effect on the time to clearance of all symptoms (Table 4). For the difference in the changes between the two groups, the time to clearance of all symptoms in the intervention group showed significant differences compared with the placebo group (95% CI, 21.17 to 43.95; *P* < 0.0001).

#### 3.1.3. Levels of IL-6, TNF-*α*, SOD, and MDA in the Peripheral Blood

The results showed that the difference in IL-6 levels before and after treatment was statistically significant between the intervention group and placebo group (adjusted difference, 6.88, 95% CI, 2.00 to 11.77; *P*=0.007); and similar results were observed for the SOD (adjusted difference, 26.14, 95% CI, 2.14 to 50.14; *P*=0.033). However, the difference in TNF-*α* levels before and after treatment was not statistically significant between the intervention group and placebo group (adjusted difference, 7.85, 95% CI, 2.10 to 17.81; *P*=0.120); and similar results were observed for the MDA (adjusted difference, 3.51, 95% CI, 2.53 to 9.55; *P*=0.248). The results are shown in Table 5.

#### 3.1.4. The Time to Disappearance of Primary Symptoms and Secondary Symptoms

**Figure 3:**
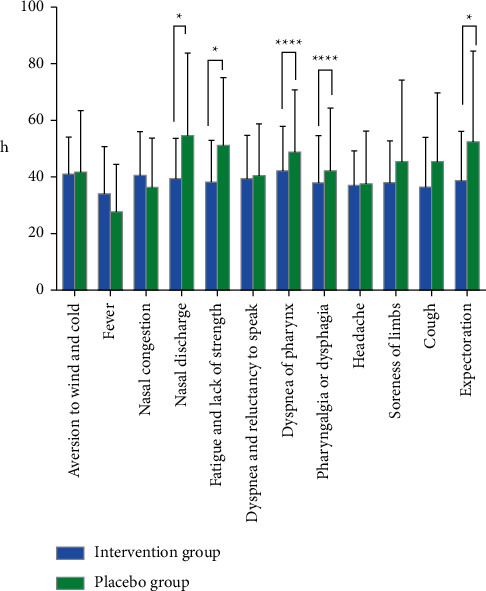
Statistical chart of the time to disappearance of primary symptoms and secondary symptoms. Graphs represent mean ± SD. ^*∗*^*P* < 0.05 and ^*∗∗∗∗*^*P* < 0.0001 compared with the intervention group.

The primary symptoms include aversion to wind and cold, fever, nasal congestion, nasal discharge, fatigue and lack of strength, dyspnea and reluctancy to speak, dryness of the pharynx, and pharyngalgia or dysphagia. The secondary symptoms include headache, limb soreness, cough, and expectoration. Ganduqing granules showed a greater effect on nasal discharge (95% CI, 3.398 to 26.88; *P*=0.0124), fatigue and lack of strength (95% CI, 2.739 to 23.10; *P*=0.0138), dryness of the pharynx (95% CI, 17.30 to 40.66; *P*=0.0001), pharyngalgia or dysphagia (95% CI, 18.03 to 43.48; *P*=0.0001), and expectoration (95% CI, 0.4415 to 27.19; *P*=0.0431) compared with the placebo group. It shortened the disappearance time of symptoms described above compared with placebo, as shown in Figure 3. However, the intervention group did not differ from the placebo group in terms of aversion to wind and cold, fever, nasal congestion, dyspnea and reluctancy to speak, headache, limb soreness, and cough.

#### 3.1.5. The Clinical Symptom Remission Rate

In the intervention group, all patients achieved clinical remission, while 25 patients achieved clinical remission in the placebo group. Thus, 100% of patients achieved clinical remission in the intervention group, whereas 82.76% in the placebo group, which showed a statistically significant difference (*P*=0.0157), as shown in Table 6.

#### 3.1.6. Change in TCM Syndrome Score

The effects of Ganduqing granules and placebo on the TCM syndrome score are shown in Table 7. TCM syndrome scores include primary symptoms and secondary symptoms, with primary symptoms calculated according to a 0–6-point scoring system and secondary symptoms calculated according to a 0–3-point scoring system. The difference in the TCM syndrome score before and after treatment was statistically significant between the intervention group and placebo group (adjusted difference, 0.87, 95% CI, 0.04 to 1.70; *P*=0.040).

## 4. Discussion

Many randomized controlled trials (RCTs) of TCM for the treatment of the common cold have recently been carried out in China. These RCTs provide many valuable treatment ideas and formulas. Nonetheless, there are also some disputed factors; for example, there is a lack of uniformity in common cold syndrome types and in the dosages of Chinese patent medicine prescriptions, and the compatibility of some drugs is unknown. There are currently no guidelines to provide accurate treatment recommendations, which can only be based on previous studies. Previous small-sample clinical trials have demonstrated the effectiveness of Ganduqing granules for the common cold, [36, 37] but there are no high-quality RCTs in the database. To investigate the efficacy and safety of Ganduqing granules in the treatment of the common cold, we conducted a high-quality RCT. Our results demonstrated that Ganduqing granules were significantly better than the placebo group in shortening the time to clearance of all symptoms. In addition, it showed a trend of greater effect on nasal discharge, fatigue and lack of strength, dryness of the pharynx, pharyngalgia or dysphagia, and expectoration.

Ganduqing granules are a type of TCM compound preparation that has an invigorating Qi and strengthening body resistance effect. It can relieve the symptoms of the common cold, according to our preliminary small-sample clinical research. It is composed of Radix Astragali and Rhizoma Belamcandae. Radix Astragali has the function of invigorating Qi, [55] and its combination with Rhizoma Belamcandae can be useful in the symptomatic relief of pharyngeal [43] and upper respiratory symptoms [36]. Radix Astragali has been found to possess anti-inflammatory and antioxidant stress effects, and our previous research confirmed that the active ingredients of Radix Astragali can reduce airway inflammation by regulating TLR4/MyD88/NF-*κ*B signaling pathway [39]. In addition, anti-inflammatory and antiviral properties are found in Rhizoma Belamcandae and it exerts anti-inflammatory effects by regulating MAPK and NRF2 pathways [56, 57].

Network pharmacology is an efficient strategy to predict the potential targets of TCM in the treatment of the diseases. Ganduqing granules have the characteristics of multiple component compatibility and multiple target systems, which is consistent with the research ideas of network pharmacology. According to the results of network pharmacology, oxidative stress and inflammatory signaling pathways were defined as the core pathways in the treatment of common cold by Ganduqing granules. Research showed that the inhibition of oxidative stress and reduction of inflammatory factors could prevent the common cold [58]. TNF-*α* and IL-6 may contribute to the pathophysiology of airway inflammation [59, 60]. Therefore, we examined the levels of IL-6, TNF-*α*, SOD, and MDA in the peripheral blood in our research [61]. The two treatments, Ganduqing granules and placebo, reduced the levels of IL-6, TNF-*α*, and MDA. In addition, the SOD levels increased after treatment in the intervention group and placebo group. This may have been partly because some common cold cases may resolve spontaneously [62]. Our results indicated that the changes in the levels of TNF-*α* and MDA did not show significant differences between the intervention and placebo groups. However, the decrease of IL-6 levels in the intervention group was more significant than that in the placebo group; similar results were observed for the SOD. This illustrated that Ganduqing granules have a more prominent effect on the common cold to some extent. As a result, Ganduqing granules may act as an effective agent through multiple targets in the prevention and treatment of the common cold. The benefits of traditional Chinese medicine are reflected in this possible multitarget impact.

The common cold of Qi-deficiency syndrome is a type of common cold. According to the theory of traditional Chinese medicine, the Qi is stored in the body, and the evil cannot invade. When evil is gathered together, its Qi must be depleted. Qi-deficiency is the main pathogenesis of the common cold in TCM. Qi-deficiency may increase the susceptibility of the body to external invasion by pathogens, causing disorders of the blood, body fluids, and metabolism, as well as dysfunction of the viscera, which may predispose to the common cold. Therefore, we used the TCM syndrome score to evaluate the efficacy of Ganduqing granules in treating the common cold of Qi-deficiency syndrome. The results showed that the decrease of TCM syndrome score in the intervention group was more significant than that in the placebo group.

Two participants in the placebo group suffered from nausea, and the intervention group did not report any adverse effects. It may be related to maltodextrin in the placebo, and some subjects reported gastrointestinal symptoms after maltodextrin administration [63]. The nausea resolved spontaneously within 24 hours, which demonstrated that Ganduqing granules are safe for patients.

In this study, considering the course of the common cold ranges from 3 to 7 days, we selected the onset time within 48 hours as the inclusion standard. In view of the high docility of adult patients and the large number of chronic underlying diseases in elderly patients, we selected patients aged 18–65 for the trial. We also quantified the symptoms of the common cold of Qi-deficiency syndrome in TCM and evaluated the efficacy of Ganduqing granules in the treatment of the common cold, with the goal of minimizing baseline disparities among patients for clinical evaluation.

This multicenter, randomized, double-blind, placebo-controlled study aimed to clarify the efficacy of Ganduqing granules by comparing the intervention group with the placebo group. To promote appropriate high-quality methodology and strict quality control, the study was strictly designed in accordance with the CONSORT statement [50]. Moreover, it followed ethical principles and strictly implements management, monitoring, and reporting standards. In this trial, we provided a detailed description of the methods of recruitment, randomization, allocation concealment, and data collection that were used in this clinical study. We also reported the drug intervention according to the CONSORT Extension for Chinese Herbal Medicine Formulas 2017: Recommendations, Explanation, and Elaboration [64]. The results of this study provided scientific and rigorous evidence for the clinical efficacy of Ganduqing granules in the treatment of the common cold.

This clinical trial has several advantages. First, this study was designed as an RCT, which is considered to be the gold standard for evaluating the effectiveness and safety of drugs. What's more, the study was carried out using multicenter evaluations at four hospitals in Sichuan, which reduced the risk of selection bias to a certain extent. Third, this study adopted a competitive enrollment method to ensure that the subjects enrolled at each center were from the same time period, avoiding bias related to the enrollment time and improving the study efficiency. Fourth, since clinical symptoms were accompanied by a certain degree of subjectivity, we used the TCM syndrome score criteria to ensure scientific objectivity. Finally, researchers at each center obtained appropriate qualifications and training before the start of this clinical trial.

Despite the above-listed strengths, the design of our study has some potential limitations, such as the short duration of treatment, and there was no long-term follow-up of patients after treatment. In addition, multicenter locations were not selected nationwide, which may reduce the representativeness of the sample.

## 5. Conclusion

In conclusion, the clinical efficacy and safety of Ganduqing granules in treating the common cold were observed in this trial based on network pharmacology. The results demonstrated that Ganduqing granules could improve common cold symptoms, shorten the disease course, attenuated inflammation and oxidative stress, and provided objective evidence for the efficacy and safety of a Chinese herbal medicine in treating the common cold.

## Figures and Tables

**Table 1:** Agents used in Western medicine to treat the common cold.

Type of treatment	Therapeutic agent
Antipyretic analgesics [9]	Acetaminophen [10], aspirin [11], ibuprofen [12], nimesulide [12]
Antihistamines [13, 14]	Chlorpheniramine maleate [15], clemastine fumarate [16]
Decongestants [17]	Pseudoephedrine [18], xylometazoline [19]
Antitussives	Ipratropium bromide [20], guaifenesin [21], codeine [22], dextromethorphan [23]
Expectorants	Bromhexine [24], carbocysteine [25], N-acetylcysteine [26, 27]
Trace elements	Zinc [28–31], vitamin D [32], vitamin C [33]
Others	Probiotics [34]

**Table 2:** Multitarget mechanisms underlying pharmacological effects of Ganduqing granule components.

Chinese name	Latin name	Amount (g)	Pharmacological effects relevant to the common cold
Huangqi	Radix Astragali	20	Anti-inflammatory [39, 40], antioxidation [41], antiallergic [42], immune regulation [40]
Shegan	Rhizoma Belamcandae	10	Anti-inflammatory [43], antibacterial [44], antiviral [45], antioxidation [43], immune regulation [46]

**Table 3:** The baseline characteristics of participants.

Group	Intervention group (*n* = 31) (mean ± SD)	Placebo group (*n* = 29) (mean ± SD)
Male (%)	23 (74%)	21 (72%)
Female (%)	8 (26%)	8 (28%)
BMI (kg/m^2^)	22.96 ± 3.096	21.85 ± 3.052
Age (year)	40.39 ± 17.16	37.69 ± 16.24
Height (cm)	163 ± 6.723	163.5 ± 6.780
Weight (kg)	61.05 ± 8.83	58.51 ± 9.542
Body temperature (°C)	37.34 ± 0.7051	37.49 ± 0.7040
Breath (/min)	17.16 ± 3.551	16.93 ± 2.975
Heart rate (/min)	81 ± 12.02	78.62 ± 9.796
Diastolic pressure (mmHg)	78.48 ± 9.825	79.86 ± 10.89
Systolic pressure (mmHg)	114 ± 13.43	110.7 ± 22.18
Time from onset to enrollment (*h*)	35.61 ± 9.025	33 ± 11.62
IL-6	15.69 ± 30.64	14.85 ± 10.42
TNF-*α*	156.5 ± 118.0	153.3 ± 120
SOD	73.45 ± 89.64	68.79 ± 46.76
MDA	20.13 ± 20.36	19.56 ± 13.40
TCM syndrome score	20.65 ± 6.243	25.41 ± 7.023

**Table 4:** The time to clearance of all symptoms (*h*).

Group	Time to clearance of all symptoms (mean ± SD)	*P* value
Intervention group (*n* = 31)	46.06 ± 14.23	<0.0001
Placebo group (*n* = 29)	78.62 ± 28.07	

**Table 5:** Levels of IL-6, TNF-*α*, SOD, and MDA in the peripheral blood.

Indicators	Group	Means ± standard deviation (the difference between the values measured before and after treatment)	Least squares mean (the difference between the values measured before and after treatment) and 95% CI	*t*-test	
				*t* value	*P* value
IL-6	Intervention group	12.62 ± 30.04	6.88 (2.00–11.77)	2.83	0.007
	Placebo group	4.02 ± 10.20			

TNF-*α*	Intervention group	134.35 ± 100.59	7.85 (2.10–17.81)	1.58	0.120
	Placebo group	126.23 ± 104.11			

SOD	Intervention group	36.18 ± 55.49	26.14 (2.14–50.14)	2.19	0.033
	Placebo group	11.68 ± 30.33			

MDA	Intervention group	6.12 ± 16.19	3.51 (2.53–9.55)	1.17	0.248
	Placebo group	4.28 ± 9.13			

**Table 6:** The clinical symptom remission rate.

Group	Number of all symptom remission cases	Number of symptom nonremission cases	Clinical symptom remission rate (%)	*P* value
Intervention group	31	0	100	0.0157
Placebo group	25	4	82.7	

**Table 7:** Change in TCM syndrome score.

Indicators	Group	Means ± standard deviation (the difference between the values measured before and after treatment)	Least squares mean (the difference between the values measured before and after treatment) and 95% CI	*t*-test	
				*t* value	*P* value
TCM syndrome score	Intervention group	24.55 ± 5.23	0.87 (0.04–1.70)	2.11	0.040
	Placebo group	24.14 ± 5.70			

## Data Availability

The data are not publicly available due to privacy or ethical restrictions.

## Supplementary Materials

Supplementary Materials Supplemental File 1: the study program details. Supplemental File 2: specific scoring rules of TCM.
