# Supplementary material for: Efficacy and Safety of Ganduqing Granules in Treating the Common Cold: A Multicenter, Randomized, Double-Blind, Placebo-Controlled Trial
Source: Evid Based Complement Alternat Med. 2022 Jun 9;2022:5105503. doi: 10.1155/2022/5105503 (PMC9203204; doi:10.1155/2022/5105503)
Supplement: Supplementary Materials — Supplemental File 1: the study program details. Supplemental File 2: specific scoring rules of TCM. [file 5105503.f1.zip › 5105503.f1/Supplementary File 1. The study program details.docx]

**Supplementary File 1.** The study program details.

|  | **STUDY PERIOD** | | | | | | | |
| --- | --- | --- | --- | --- | --- | --- | --- | --- |
|  | **Enrollment** | **Allocation** | **Post-allocation** | | | | | |
| **TIMEPOINT** | **Day-1** | **Day0** | **Day1** | **Day2** | **Day3** | **Day4** | **Day5** | **Day6** |
| ENROLMENT: |  | | | | | | | |
| Eligibility screen | **√** |  |  |  |  |  |  |  |
| Informed consent | **√** |  |  |  |  |  |  |  |
| Demographic information | **√** |  |  |  |  |  |  |  |
| Disease situation | **√** |  | **√** |  |  |  |  | **√** |
| Physical examination | **√** |  | **√** |  |  |  |  | **√** |
| Medical history | **√** |  |  |  |  |  |  |  |
| Comorbidity | **√** |  |  |  |  |  |  |  |
| Concomitant treatment | **√** | **√** | **√** | **√** | **√** | **√** | **√** | **√** |
| Family medical history | **√** |  |  |  |  |  |  |  |
| Allergic history | **√** |  |  |  |  |  |  |  |
| Allocation |  | **√** |  |  |  |  |  |  |
| INTERVENTIONS: |  | | | | | | | |
| Ganduqing granules |  |  | **√** | **√** | **√** | **√** | **√** |  |
| Placebo |  |  | **√** | **√** | **√** | **√** | **√** |  |
| ASSESSMENTS: |  | | | | | | | |
| Clinical sign and symptom score | **√** |  | **√** | **√** | **√** | **√** | **√** | **√** |
| Symptom duration |  |  | **√** | **√** | **√** | **√** | **√** | **√** |
| Levels of IL-6 | **√** |  |  |  |  |  |  | **√** |
| Levels of TNF-α | **√** |  |  |  |  |  |  | **√** |
| Levels of SOD | **√** |  |  |  |  |  |  | **√** |
| Levels of MDA | **√** |  |  |  |  |  |  | **√** |
| Routine blood test | **√** |  |  |  |  |  |  | **√** |
| Urinalysis | **√** |  |  |  |  |  |  | **√** |
| Routine stool test | **√** |  |  |  |  |  |  | **√** |
| Liver function test | **√** |  |  |  |  |  |  | **√** |
| Renal function test | **√** |  |  |  |  |  |  | **√** |
| Chest X-ray | **√** |  |  |  |  |  |  |  |
| Electrocardiogram | **√** |  |  |  |  |  |  | **√** |
| Adverse event record |  |  | **√** | **√** | **√** | **√** | **√** | **√** |
| Drug distribution |  | **√** |  |  |  |  |  |  |
| Drug recycling and count |  |  |  |  |  |  |  | **√** |
