# Supplementary material for: Efficacy and Safety of Ganduqing Granules in Treating the Common Cold: A Multicenter, Randomized, Double-Blind, Placebo-Controlled Trial
Source: Evid Based Complement Alternat Med. 2022 Jun 9;2022:5105503. doi: 10.1155/2022/5105503 (PMC9203204; doi:10.1155/2022/5105503)
Supplement: Supplementary Materials — Supplemental File 1: the study program details. Supplemental File 2: specific scoring rules of TCM. [file 5105503.f1.zip › 5105503.f1/Supplementary File 2. Specific Scoring Rules of TCM..docx]

**Supplementary File 2.** Specific Scoring Rules of TCM.

| **Symptom** | **Score** | | | | **Symptom duration**  **(h)** |
| --- | --- | --- | --- | --- | --- |
| **Primary** |  | **2** | **4** | **6** |  |
| Aversion to wind and cold | None | Mild aversion to wind | Aversion to wind and relief with additional clothing | Aversion to cold and no effect of additional clothing |  |
| Fever | None | **37.3~37.9℃** | **38.0~38.4℃** | **38.5℃~39℃** |  |
| Nasal congestion | None | Nasal congestion  and the nasal sound is heavy | Nasal congestion on and off | Persistent nasal congestion |  |
| Nasal discharge | None | Mild  nasal discharge | Nasal discharge on and off | Persistent  nasal discharge |  |
| Fatigue and lack of strength | None | Mild  fatigue | Severe  fatigue, reluctantly tolerating daily activities | Myasthenia of limbs and inability to tolerate daily activities |  |
| Shortness of breath and reluctant  to speak | None | Shortness of breath after exercise and reluctance  to speak | Shortness of breath after a little movement and reluctance  to speak | Myasthenia of limbs and inability to support daily activities |  |
| Dryness of pharynx | None | Mild dry pharynx | Dry pharynx with thirst, relief with drinking | Dry pharynx with thirst, no relief with drinking |  |
| Pharyngalgia or dysphagia | None | Mild pharyngalgia, congestion of throat and no effects on swallowing | Severe pharyngalgia, obvious when swallowing and congestion of pharynx mucosa | Pharyngalgia affecting rest, congestion of pharynx mucosa and diffuse uvula hyperemia and edema |  |
| **Secondary** |  | **1** | **2** | **3** |  |
| Headache | None | Seldom | Occasionally | Frequently |  |
| Soreness of limbs | None | Mild | Moderate | Severe |  |
| Cough | None | Seldom | Occasionally | Frequently |  |
| Expectoration | None | Seldom | Occasionally | Frequently |  |
| Tongue appearance and pulse condition | No score, but the tongue appearance and pulse condition of the participants before and after treatment should be recorded in detail. | | | |  |
| Total score |  | | | |  |
